# Inter-individual variation in DNA methylation is largely restricted to tissue-specific differentially methylated regions in maize

**DOI:** 10.1186/s12870-017-0997-3

**Published:** 2017-02-23

**Authors:** Massimiliano Lauria, Rodrigo Antonio Echegoyen-Nava, Dalia Rodríguez-Ríos, Silvio Zaina, Gertrud Lund

**Affiliations:** 1 Consiglio Nazionale delle Ricerche, Istituto di Biologia e Biotecnologia Agraria, I-20133 Milan, Italy; 2 Gertrud Lund, Department of Genetic Engineering, CINVESTAV - Unidad Irapuato, Km. 9.6 Libramiento Norte Carretera Irapuato-Leon, Apdo. Postal 629, C. P. 36500 Irapuato, GTO Mexico; 3 Department of Medical Sciences, Division of Health Sciences, León Campus, University of Guanajuato, Guanajuato, Mexico

**Keywords:** Inter-individual variation in methylation, Tissue-specific, CG and non-CG methylation, Class II transposable elements, Endosperm

## Abstract

**Background:**

Variation in DNA methylation across distinct genetic populations, or in response to specific biotic or abiotic stimuli, has typically been studied in leaf DNA from pooled individuals using either reduced representation bisulfite sequencing, whole genome bisulfite sequencing (WGBS) or methylation sensitive amplified polymorphism (MSAP). The latter represents a useful alterative when sample size is large, or when analysing methylation changes in genomes that have yet to be sequenced. In this study we compared variation in methylation across ten individual leaf and endosperm samples from maize hybrid and inbred lines using MSAP. We also addressed the methodological implications of analysing methylation variation using pooled versus individual DNA samples, in addition to the validity of MSAP compared to WGBS. Finally, we analysed a subset of variable and non-variable fragments with respect to genomic location, vicinity to repetitive elements and expression patterns across leaf and endosperm tissues.

**Results:**

On average, 30% of individuals showed inter-individual methylation variation, mostly of leaf and endosperm-specific differentially methylated DNA regions. With the exception of low frequency demethylation events, the bulk of inter-individual methylation variation (84 and 80% in leaf and endosperm, respectively) was effectively captured in DNA from pooled individuals. Furthermore, available genome-wide methylation data largely confirmed MSAP leaf methylation profiles. Most variable methylation that mapped within genes was associated with CG methylation, and many of such genes showed tissue-specific expression profiles. Finally, we found that the *hAT* DNA transposon was the most common class II transposable element found in close proximity to variable DNA regions.

**Conclusions:**

The relevance of our results with respect to future studies of methylation variation is the following: firstly, the finding that inter-individual methylation variation is largely restricted to tissue-specific differentially methylated DNA regions, underlines the importance of tissue-type when analysing the methylation response to a defined stimulus. Secondly, we show that pooled sample-based MSAP studies are methodologically appropriate to study methylation variation. Thirdly, we confirm that MSAP is a powerful tool when WGBS is not required or feasible, for example in plant species that have yet to be sequenced.

**Electronic supplementary material:**

The online version of this article (doi:10.1186/s12870-017-0997-3) contains supplementary material, which is available to authorized users.

## Background

In plants cytosine methylation occurs at symmetric 5′-CpG-3′ dyads (CG) and 5′-CpHpG-3′ (CHG; H is A, C or T) triads, in addition to asymmetric 5′-CpHpH-3′ (CHH) triads [1–3]. In each case, methylation is controlled by distinct DNA methyltransferases. In *Arabidopsis thaliana*, the main CG, CHG and CHH methylases are METHYLTRANSFERASE1 (MET1), CHROMOMETHYLASE 3 (CMT3) and CHROMOMETHYLASE 2 (CMT2) or DOMAINS-REARRANGED METHYLTRANSFERASE 2 (DRM2), respectively. In maize, the corresponding homologs are ZMET1 and ZMET2 or 5, CMT2 is absent and ZMET3 [4–10]. In addition, the DOMAINS-REARRANGED METHYLTRANSFERASE 2 (or ZMET3 in maize) plays an important role in the RNA-directed DNA methylation pathway [4, 9, 11], first discovered in tobacco plants [12], which culminates with de novo methylation of cytosine in CG, CHG and CHH contexts in response to small RNA signals (reviewed in [13]).

The genome-wide distribution of DNA methylation has been detailed both in arabidopsis [14, 15] and agronomically important plants such as rice, maize, soybean, cassava, soybean, common bean, wheat and cotton [16–23]. Collectively, these studies show that the bulk of DNA methylation is located within transposable elements (TEs), underlining its important and well-characterized function - proposed several years ago - in regulating TE activity [24, 25]. In addition, those data also uncovered the prevalence of CG methylation within the gene-body.

To date, nearly all genome-wide methylation studies of natural variation in DNA methylation, either within a genetically identical population following several generations, or across distinct genetic populations or tissue-types, compare average DNA methylation states of pooled individuals or less than 2 individuals per generation [18, 25–36]. However, the few studies that take inter-individual variation into account, show that both natural and stress-induced methylation responses are heterogeneous across individuals and can vary between developmental stages [37–42]. All the aforementioned studies were performed using the methylation sensitive amplified polymorphism (MSAP) technique. Although this technique only surveys the methylation state of a defined restriction enzyme site that is sensitive to DNA methylation (*e.g. Hpa*II), it does give a reliable readout of the genome-wide methylation state. As an example, we demonstrated that the 13% reduction in DNA methylation in maize endosperm relative to leaf and embryo tissues largely resulted from maternal hypomethylation [42], results that were subsequently confirmed by high-throughput bisulfite sequencing of arabidopsi*s*, rice, sorghum, maize and castor bean genomes [34, 43–47].

Currently scarce information is available regarding inter-individual methylation variation (ii-MV) across genetically identical progeny and whether such variation differs across plant tissues. Given the lack of such studies, we analysed methylation profiles of ten individual leaf and endosperm tissues derived from single cobs of two hybrid and one inbred line by MSAP. Furthermore, since pooled samples have been used in the majority of DNA methylation variation studies, we addressed the important methodological issue of whether individual samples better reflect methylation variation compared to pooled samples. Our data reveal that ii-MV is readily detected in both leaf and endosperm tissue, but largely restricted to tissue-specific differentially methylated regions (tDMRs). We find that the majority of such variation is detectable by analysis of pooled samples and show that MSAP represents a reliable alternative to WGBS for analysing methylation variation.

## Results

### Characterization of inter-individual methylation variation (ii-MV) in maize endosperm and leaf using MSAP

MSAP was employed to characterize ii-MV. This technique is a modification of AFLP (**A**mplified **F**ragment **L**ength **P**olymorphism), which is based on random amplification of restriction fragments typically generated by digestion of genomic DNA with *Eco*RI and *Mse*I restriction enzymes [48]. In MSAP, *Mse*I is replaced by *Hpa*II, which cleaves CCGG sites, unless one or both cytosines are methylated on both strands [49]. Adaptors are ligated to digested restriction sites and resulting fragments are subsequently amplified in two consecutive PCR reactions with primers complementary to core sequence of adaptors and recognition sites of restriction enzymes. Typically, the number of selective nucleotides added to the primers at 3′ ends is increased in the second amplification reaction. In addition, one primer is radioactively labelled to enable visualization of restriction fragments by autoradiography.

**Fig. 1:**
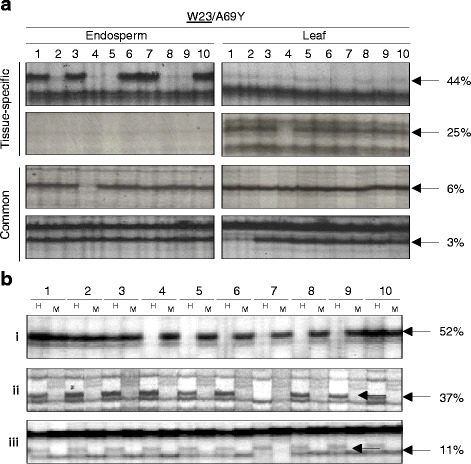
MSAP analysis of individual tissues derived from a single cob of **W23**/A69Y hybrid (the egg donor of the cross is underlined). **a** MSAP analysis of 10 individual two-week-old leaves and 15 DAP endosperms; *arrows* indicate tissue-specific and common MSAP fragments that vary across individual endosperm and leaves (the two *upper* and two *lower* panels, respectively); **b** MSAP analysis of 10 individual endosperms using either *Hpa*II (H) or *Msp*I (M) in the initial restriction digest; panels shows examples of inter-individual variation in CG methylation (i and ii) and CHG methylation, or CG and CHG methylation (iii)

Ten individual endosperms harvested 15 days after pollination (DAP) and ten 14-day-old leaves (**W23**/A69Y, the seed donor is in bold) were analysed by MSAP and AFLP using 12 and 10 selective primer combinations, respectively. In either case, tissue samples were derived from a single hybrid cob. A total of 13 (69/526) and 3% (14/440) of endosperm and leaf MSAP fragments, respectively, showed variation across individuals (Additional file 1: Table S1). The higher number of total MSAP fragments in endosperm versus leaf (*i.e*. 526 and 440, respectively) was expected given that the former is hypomethylated relative to leaf [42]. Conversely, no variation was detected by AFLP (results not shown). Further MSAP analyses of individual endosperms and leaves from the **Mo17**/B73 hybrid, in addition to individual endosperms from the A69Y inbred line, revealed that ii-MV did not differ significantly, neither between genetic background, nor between inbred and hybrid lines (Additional file 1: Table S1). Subsequently, we scored ii-MV of tissue-specific or common MSAP fragments of the two hybrid crosses; i.e. of MSAP bands detected in one or both tissues, respectively (Fig. 1a and Table 1). In either hybrid, ii-MV was significantly increased of tissue-specific compared to common MSAP band (*p* < 0.0005 and *p* < 0.0001, respectively). However, neither common nor tissue-specific MSAP fragments showed significant differences in ii-MV between tissues.

**Table 1:** Inter-individual variation in methylation of common and tissue-specific MSAP fragments

		Total^a^		Variable^b^		% Variable	
		C^c^	Ts^d^	C^c^	Ts^d^	C^c^	Ts^d^
**W23**/A69Y	Endosperm	428	98	26	43	6	44
	Leaf	428	12	11	3	3	25
**Mo17**/B73	Endosperm	507	155	30	55	6	35
	Leaf	507	21	15	5	3	24

^a^Sum of MSAP fragments that lacked or showed variation in DNA methylation in endosperm or leaf tissue using 12 selective primer combinations

^b^MSAP fragments that showed variation in methylation between individual endosperms or leaves

^c^C = Common MSAP fragments (*i.e*. fragments detected in both leaf and endosperm)

^d^Ts = Tissue-specific MSAP fragments (*i.e*. fragments detected either in leaf or endosperm)

Next, we assessed whether ii-MV occurred preferentially of cytosines in a CG or CHG context by comparing MSAP profiles of individual endosperms from the **W23**/A69Y hybrid using either *Hpa*II or its isoschizomer *Msp*I in the initial restriction digest. These restriction enzymes differ in their sensitivity to methylation of the CCGG recognition site: *Hpa*II is sensitive to methylation of either cytosine residues, but is insensitive to hemi-methylation of the external cytosine residue, whereas *Msp*I is sensitive to hemi- or complete methylation of the external cytosine. We found that 89% of ii-MV occurred in a CG context; i.e. variation was only detected following *Hpa*II, but not *Msp*I digestion (Fig. 1b, panels i and ii). In 37% (28/75) of such cases, no fragment was detected with *Msp*I (panel ii). This suggested either that the external cytosine residue of the CCGG recognition site was hemi-methylated, or the presence of an internal *Hpa*II site that was methylated in a CG context only. The latter explanation is likely given that ~20% of MSAP fragments have internal CCGG sites [42]. In contrast, only 11% (8/75) of ii-MV occurred exclusively in a CHG context, or in both a CG and CHG context; i.e. band absence following both *Hpa*II and *Msp*I digestion (Fig. 1b, panel iii).

To understand whether these profiles reflected context-dependent endosperm-specific hypomethylation, we performed an in vitro methyl-accepting assay on 15 DAP endosperms and 14-day-old leaf tissues from the W23 inbred line. This assay exploits the ability of bacterial DNA *Hpa*II and *Msp*I methylases to methylate the internal and external cytosine of unmethylated CCGG sequences, respectively [50]. In addition, total CG methylation was measured with the *Sss*I methylase, which methylates cytosines in CpG dinucleotides independent of sequence context. Although CG methylation levels were increased twofold relative to CHG methylation, the methylation level of either was reduced fivefold in endosperm compared to leaf (Table 2). This contrasts with a comparative Methyl-seq analysis of 12 DAP endosperm and leaf tissue from the B73 inbred line where only CHG methylation differed significantly between leaf and endosperm tissues [34]. This discrepancy may result either from inbred or developmental-specific differences in methylation, or alternatively from technical issues related to amplification of bisulfite-treated DNA such as selective enrichment of unmethylated alleles [51].

**Table 2:** Methyl-accepting assay of endosperm and leaf DNA

		Target/haploid genome (x10^6^)^a^	
Methylase	Target sequence^b^	Endosperm^c^	Leaf^c^
*Hpa*II	CCGG	0.34 ± 0.12	0.071 ± 0.016
*Msp*I	CCGG	0.64 ± 0.24	0.16 ± 0.024
*Sss*I	CG	1.94 ± 0.51	0.41 ± 0.25

^a^Mean ± SD; *n* = 6 and *n* = 5 of endosperm and leaf, respectively

^b^Underlined cytosine indicates target of methylase

^c^All differences between groups (i.e. between tissues with same enzyme treatment and between enzyme treatments within tissues) were statistically significant (*p* < 0.05)

### The methylation state of pooled samples largely reflects the predominant methylation profile across individual samples

**Fig. 2:**
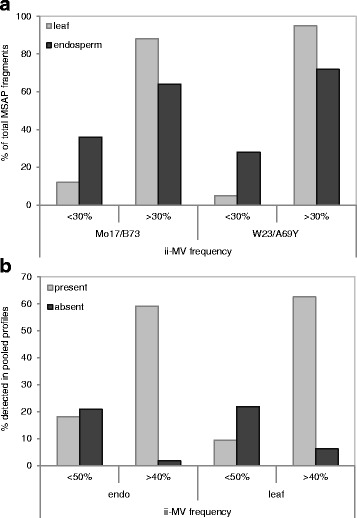
Frequency of inter-individual methylation variation (ii-MV). **a** the percentage of endosperm and leaf MSAP fragments with ii-MV frequencies <30 or >30%; frequencies were scored as the percentage of individuals where a band was detected/total number of individuals analysed; **b** percentage of endosperm and leaf variable MSAP fragments that were present or absent in MSAP profiles from pooled individuals; percentages were compared of ii-MV frequencies that were <50% or >40%

Given that genome-wide studies are frequently performed on DNA pooled from several individuals, we asked to what extent the methylation state of a DNA sample from pooled individuals captured ii-MV. First, we performed an MSAP experiment to detail the limits of detection of particular methylation profiles. To this end, we analysed the inbred lines Mo17 and B73 and their respective reciprocal crosses by MSAP and identified 20 MSAP fragments that were specific to the B73 inbred line. Subsequently, we scored for the presence or absence of these fragments in an “artificial” **B73**/Mo17 hybrid where the genomic contribution of B73 ranged between 10 and 100%; i.e. ten dilution series at 10% intervals that were generated by spiking B73 DNA with DNA from the Mo17 inbred line. These experiments showed that all B73-specific bands were consistently detected when the genomic contribution of B73 was higher than 30%. However, at a 10 and 20% B73 genomic contribution, the fidelity of detection dropped to 70 and 85%, respectively. Translating these results to interpreting MSAP variability data, the majority of band absence occurs when methylation levels are >90%, while methylation levels between 30 and 90% are indistinguishable. Furthermore, these data suggest that a discrete portion of fragments that are only present in 10–30% of analysed individuals could represent profiles that cannot be accurately determined by MSAP. This prompted us to analyse the frequency of ii-MV in leaf and endosperm tissues of either hybrid line. This frequency was calculated as number of individuals where a band is present divided by total number of individuals analysed. In either tissue, the bulk of variable fragments were detected in more than 30% of individuals analysed - i.e. 88 and 64% of **Mo17**/B73 leaf and endosperm MSAP fragments, respectively; the corresponding values were 95 and 72% in the **W23**/A69Y hybrid (Fig. 2a). This indicated that most MSAP ii-MV represented true biological variation. Next, we assessed whether the most abundant methylation state observed across individual leaves and endosperms from the **Mo17**/B73 hybrid was accurately captured in a pooled sample of either tissue. As expected, the majority of endosperm and leaf MSAP fragments that were present in less than 50% of individuals were associated with band absence in the pooled profile, while MSAP fragments that were present in more than 40% of individuals showed the opposite behaviour (Fig. 2b). In total, these fragments accounted for 84 and 80% of leaf and endosperm variable MSAP fragments, respectively. Of the remaining leaf and endosperm MSAP fragments that deviated from expected profiles, 60 and 90%, respectively, represented low frequency demethylation events (*i.e*. band presence in less than 50% of individuals) (Fig. 2b). Taken together, the data suggest that MSAP analysis of pooled samples is less effective in capturing low, than high-frequency demethylation events across individuals.

### Validation of MSAP data

We excised a total of 106 MSAP fragments that either lacked or showed ii-MV in the **Mo17**/B73 hybrid. Following sequence analysis, 26 non-variable and 28 variable fragments reached our stringent criteria for further analysis (see methods). We also identified six variable and eight non-variable previously isolated MSAP fragments [42] that showed ii-MV in the **W23**/A69Y hybrid. Using selected fragments as probes in Southern blot analysis we confirmed that endosperm-specific variable MSAP fragments showed reduced levels of both CG and CHG methylation in endosperm relative to leaf tissue, while these profiles were largely identical of a non-variable fragment (Additional file 2: Figure S1a, compare tissue-specific *Hpa*II and *Msp*I profiles). In addition, we compared ii-MV in CG and CHG context of two variable fragments and one non-variable MSAP fragment using the methylation-sensitive restriction enzymes *Hpa*II and *Msp*I, respectively (Additional file 2: Figure S1b). Both variable MSAP fragments showed ii-MV in a CG context, while variation in CHG context was restricted to the variable PMP2 band. Conversely, no evidence of inter-individual variation was observed following digestion with the *EcoR*I and *EcoR*V restriction enzymes that are generally considered methylation-insensitive (Additional file 2: Figure S1b). Likewise, no inter-individual variation was detected of the non-variable fragment, neither with methylation-sensitive nor methylation-insensitive restriction enzymes.

We also validated the methylation states of variable and non-variable *Hpa*II sites predicted by MSAP using the publicly available WGBS data from leaf tissue of B73 and Mo17 inbred lines [32]. To this end, we successfully mapped 17/26 non-variable and 24/28 variable MSAP fragments to unique regions of the B73 inbred line genome (B73 RefGen_V4) (Additional file 3) and recovered the methylation state of each *Hpa*II sites. The remaining MSAP fragments, showed partial or ambiguous overlap to the reference genome and/or lacked methylation data. Next, we predicted the methylation state of mapped variable and non-variable *Hpa*II sites in leaf based on the presence or absence of MSAP fragments across endosperm and leaf individuals (Additional file 4: Figure S2a). Most non-variable fragments (94%) were detected in both tissues - and in all individuals - and were thus predicted to be unmethylated in both leaf and endosperm. Conversely, the non-variable fragment that was specifically detected in endosperm samples was considered methylated in leaf. Using similar arguments, 79% of variable fragments (*i.e*. 67 + 8 + 4%) were expected to show some degree of methylation in leaf, while the remaining 21% were predicted to lack methylation in this tissue (Additional file 4: Figure S2a). Overall, 85% of the predicted methylation states of individual *Hpa*II sites were confirmed in WGBS data of cytosine methylation in a CG context (Additional file 4: Figure S2b). In particular, the predicted unmethylated state was confirmed - in at least one of the two inbred lines- of 88 (15/17) and 100% (5/5) of non-variable and variable fragments, respectively; the corresponding percentages for the predicted methylated state were 100 (1/1) and 79% (15/19), respectively. With respect to CHG methylation, we found that fewer non-variable and variable *HpaII* sites were associated with CHG methylation. In addition, CHG methylation levels were significantly lower that CG methylation levels in both the B73 and Mo17 inbred line (*p* < 0.0028 and 0.0003, respectively) (Additional file 4: Figure S2c).

Collectively, these data confirmed that ii-MV was largely restricted to tDMRs and preferentially associated with CG methylation. Conversely the analysed non-variable regions were largely unmethylated in either tissue.

### Characteristics of isolated variable and non-variable fragments

**Fig. 3:**
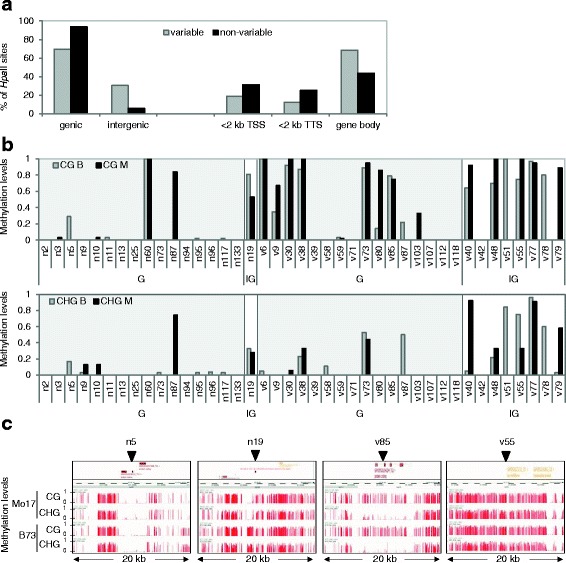
Characteristics of variable and non-variable *Hpa*II sites. **a** genomic distribution of variable and non-variable *Hpa*II sites; genic regions were defined as being within 2 kb up or downstream, respectively of transcriptional start (TSS) and termination sites (TTS) annotated in maize B73 RefGen_V4; intergenic regions were defined as being more than 2 kb up or down-stream, respectively of TSS and TTS; **b** levels of CG and CHG methylation of variable (v) and non-variable (n) *Hpa*II sites located within genic (G) and intergenic (IG) regions (*light grey* and no shading, respectively); the Y-axis indicates methylation levels between 0 and 1 (0 and 100% methylation, respectively) derived from WGBS of B73 (B) and Mo17 (M) leaf tissue [32]; **c** illustrative examples of CG and CHG methylation levels of genomic regions located 10 kb up- and downstream of non-variable and variable *Hpa*II sites located either within genic (n5 and v85) or intergenic (n19 and v55) regions; *arrowheads* indicate positions of variable and non-variable *Hpa*II sites

Most variable and non-variable *Hpa*II sites mapped within genic regions - i.e. <2 kb up or downstream of transcriptional start and termination sites annotated in the maize B73 RefGen_V4 (TSS and TTS, respectively) - in particular within the gene-body, defined as the region between TSS and TTS (Fig. 3a). With respect to CG and CHG methylation, the latter was more abundant within intergenic regions, while CG methylation was largely restricted to genic regions, both of non-variable and variable *Hpa*II sites (Fig. 3b). Furthermore, analysis of DNA regions that extended 10 kb up- and downstream of non-variable and variable *Hpa*II sites showed that their generally unmethylated and methylated state, respectively was characteristic of an extended DNA region ranging from ~1–10 kb in size (Fig. 3c and Additional file 5: Figure S3). With respect to genes harbouring variable and non-variable *HpaII* sites, 38 (6/16) and 56% (9/16) respectively, had a paralogue in maize, while 75 and 50%, respectively, had an orthologue in *Arabidopsis thaliana*. Many of the annotated functions of these genes were related to dynamic cellular processes such as transcription and cell signalling (Additional file 6: Table S2).

We also assessed whether *Hpa*II sites that showed ii-MV tended to map closer to a repetitive DNA region. As a control group, we included non-variable *Hpa*II sites. In each case, we scored the distance to the closest repeat region and annotated both its size and classification. We found that 88% of variable *Hpa*II sites were closest to class I or II transposable elements (TEs), while the majority of non-variable *Hpa*II sites (53%) were closer to a tandem repeat (Additional file 7: Figure S4a). With respect to TE, we found that the *hAT* superfamily was the most frequent class II TE found in close proximity to - and exclusively of - variable *H*paII sites (Additional file 7: Figure S4b). However, neither the average distance to a repeat region, nor its size, differed significantly between variable and non-variable *Hpa*II sites (*p* < 0.82 and *p* < 0.11, respectively).

**Fig. 4:**
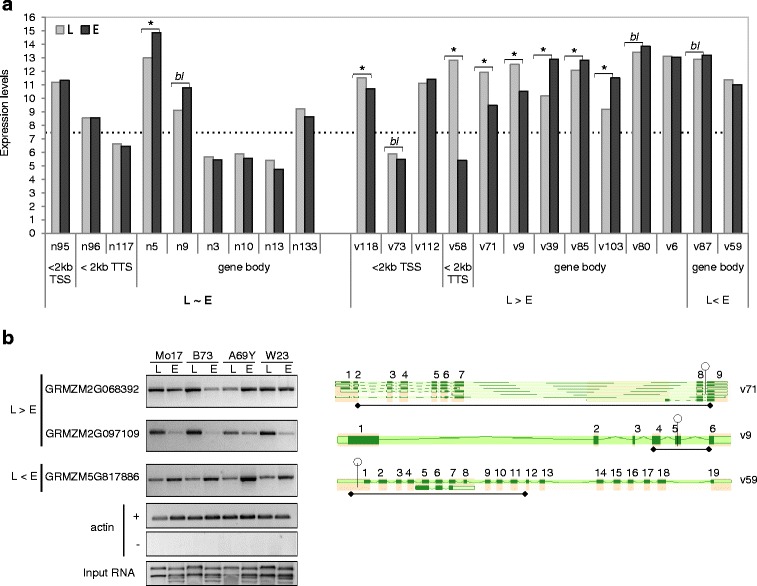
Expression profiles of genes harbouring variable and non-variable *Hpa*II sites. **a** transcription data of mixed seedling V1 stage (L) and 14 DAP endosperms (E) from ZM37 (Plant Expression Database); the *dotted line* represents the 7.6 threshold value commonly used for presence of expression calling [52]; L ~ E refers to genes that contained *Hpa*II sites that were unmethylated (detected) in either tissue, while L > E and L < E refer to *Hpa*II sites that were more or less methylated in leaf relative to endosperm, respectively (as determined by comparing endosperm and leaf MSAP profiles; see Additional file 4: Figure S2a); gene-body was defined as the region between transcriptional start and termination sites (TSS and TTS, respectively); *asterisks* indicates significant differences in expression between leaf and endosperm tissues (* = *p* < 0.05 and *bl* = borderline significance, *p* < 0.1); **b** *left panel*: RT-PCR expression profiles of three genes across leaf (L) and endosperm (E) tissues; + and – indicates presence or absence of reverse transcriptase, respectively; *right panel*: positions of variable *Hpa*II sites (open lollipops) relative to the region amplified by RT-PCR (*double*-*headed arrows*)

Finally, we analysed whether genes harbouring variable *HpaII* sites were associated with tissue-specific differences in gene expression. As a control group, we included genes harbouring non-variable *HpaII* sites. These genes will subsequently be referred to a v-genes and n-genes. The rationale for comparing expression profiles between these groups was based on their differing methylation profiles across leaf and endosperm tissue; i.e. non-variable *Hpa*II sites and their flanking regions were generally unmethylated in both tissues, while most variable sites showed tissue-specific differences in methylation. For each gene we extracted B73 transcription data of mixed seedling V1 stage and 14 DAP endosperms (ZM37-Plant Expression Database) [52]). These particular developmental stages were comparable to those used for MSAP analysis. Expression data was available for 9 n-genes and 13 v-genes and genes belonging to each group were further subdivided according to the relative methylation state of the *Hpa*II site in leaf compared to endosperm, in addition to the genic location of the *Hpa*II site (Fig. 4a). In all cases, the relative methylation states were predicted by comparing individual leaf and endosperm MSAP profiles as previously described (see Additional file 4: Figure S2a).

Overall, we found that v-genes were expressed at higher levels in leaf - but not in endosperm tissue - relative to n-genes (*p* < 0.008 and *p* < 0.123, respectively). Furthermore, the majority of v-genes (77%) showed significant (or borderline-significant) differences in expression between tissues compared to only 22% of n-genes (Fig. 4a). However, across tissue-types, we found no specific trend between methylation and expression. For example, of the seven v-genes that were more methylated within the gene-body in leaf compared to endosperm, two (v71 and v9) showed increased levels of transcription in leaf relative to endosperm tissue, while four (v39, v85, v103 and v80) showed the opposite profile (Fig. 4a). The same was true of variable *Hpa*II sites located either within 2 kb of TSS and TTS, or within gene-bodies that were more methylated in endosperm relative to leaf. To validate those database-deduced transcriptional profiles by direct expression, we designed primers pairs that spanned the variable *Hpa*II sites of MSAP fragments v71, v9 and v59 that were located within GRMZM2G068392, GRMZM2G097109 and GRMZM5G817886 (Fig. 4b). We confirmed the expression profile of the two former - i.e. their increased levels of transcription in leaf relative to endosperm tissue in the B73 inbred line. However, v59 transcription differed from the expected expression profile, since this gene showed increased levels of transcription in endosperm relative to leaf (Fig. 4b). In addition to the B73 inbred line, we also analysed RNA from Mo17, A69Y and W23 inbred lines and found that gene-specific expression profiles were largely conserved across inbred lines (Fig. 4b). The only exception was GRMZM2G068392 that showed higher levels of expression in endosperm tissue from the A69Y inbred line.

## Discussion

Our results reveal that the bulk of methylation at *Hpa*II sites (CCGG) in leaf is conserved across maize individuals germinated from single-cob seeds of either inbred or hybrid maize lines. In leaves, only ~3% of MSAP fragments showed ii-MV, which is comparable – albeit slightly higher – to the less than 1% reported of individual arabidopsis seedling leaf tissue analysed by MSAP [37]. Although we found no significant differences in the frequency of ii-MV between leaf and endosperm, the total number of variable MSAP fragments was increased four to fivefold of the latter. This finding is largely explained by the tight association between ii-MV and tDMRs and the fact that the latter are much more abundant in endosperm relative to leaf [42]. Interestingly, a study of ii-MV across genetically identical mice also found that more than 50% of variable regions overlapped with tDMRs [53].

As a consequence of the above, the bulk of variable MSAP fragments were more methylated in leaf relative to endosperm tissue. By contrast, most non-variable fragments were unmethylated; i.e. detected in both tissues. Using publicly available WGBS data of maize leaf tissue [32], we confirmed that non-variable *Hpa*II sites lacked DNA methylation, while most variable *Hpa*II sites showed varying levels of CG methylation in genic regions, or CG and CHG methylation in intergenic regions. Furthermore, these particular methylation states were generally representative of extended genomic regions ranging from ~1–10 kb in size. Taken together, the data suggest that ii-MV is preferentially associated with methylated DNA regions. In accordance, a recent analysis of single-cell methylation variation in liver tissue from the Japanese rice fish *Oryzias latipes* found that methylation-variation was increased of hyper rather than hypomethylated DNA regions [54]. Importantly, the convergence between our MSAP methylation and previously generated WGBS from leaf tissue [32], indicates that MSAP represents a reliable and representative read-out of methylation states. This suggests that MSAP represents a valuable alternative for analysing DNA methylation states, either in plant species with incomplete or no genome information, or when the optimal sample size renders WGBS (or any other next generation sequencing technique) not practical.

Similar to variation in methylation between Arabidopsis accessions and maize or soybean inbred lines [26–28, 30, 33], much ii-MV mapped within the gene-body and was largely restricted to CG methylation. A possible explanation for the absence and low levels of CHG ii-MV is that such methylation is only present transiently during transcription due to INCREASED IN BONSAI METHYLATION 1 (IBM1) activity, an H3K9 demethylase that actively prevents CMT3-mediated CHG methylation within gene bodies [55, 56]. Importantly, in this MSAP study CHG methylation was assayed in a CCG context and such methylation is largely MET1-dependent as opposed to CHG methylation in a CTG and CAG context [57]. However, given that actively transcribed genes with a high density of CTG and CAG are preferentially targeted for gene body methylation [58], we cannot exclude that methylation in those sequence contexts may be more prone to CHG ii-MV. Akin to several other studies we found a complex relationship between gene-body methylation and expression across tissues [14, 15, 18, 43, 44, 59]. Indeed, a recent study shows that the lack of gene body methylation in the angiosperm *Eutrema salsugineum* seemingly has no functional consequences with respect to transcription regulation [58].

Interestingly, a previous study of ii-MV across leaf tissue by MSAP showed that only a minority (17%) of ii-MV was conserved between two leaf developmental stages [60]. Such transient or stochastic ii-MV could have implications with regards to interpreting long-term effects of any particular stress on the epigenome or identifying epialleles generated across generations. Indeed, stress responses to phosphate starvation, heat, cold, UV or hyperosmosis have been shown to be transient or heterogeneous, both across individuals and generations [61–63]. It follows that differentially methylated regions (or differentially methylated cytosines) identified by studies performed on bulked tissues, or designed with a sub-optimal sample size, detect a combination of methylation variability that can be both transient and stable. This may be particularly relevant of low frequency demethylation events since such variation was less efficiently captured in a pooled sample by MSAP. At any rate, our data demonstrate that sample pooling can faithfully reflect at least a portion of methylation variation.

Several studies in both plants and mammals have shown that TEs exhibit both intra-individual and ii-MV [53, 64–67] and there is ample evidence of methylation variation between and within genotypes resulting from proximity to TEs [30, 32, 62–74]. In this study, we analysed whether variable *Hpa*II sites were in closer proximity to a TE compared to non-variable sites. Overall, we found no differences between these two groups, neither with respect to distance, nor size of the TE. However, we did find that variable *Hpa*II sites were preferentially located in vicinity of *hAT* superfamily of class II transposons. One obvious *caveat* of the present study is the comparatively small number of fragments yielded by the MSAP platform that showed ii-MV. Nonetheless, analysis of ii-MV in mice by whole genome bisulfite sequencing revealed only a total of 356 loci that showed ii-MV. In that study, ~15% of variable regions were associated with Endogenous retroviruses (ERV), a class I TE. Such data warrant further studies on the relevance of TE on ii-MV following specific environmental or developmental stimuli.

## Conclusions

Our data suggest that ii-MV is largely restricted to tDMRs. Importantly, we show that sample pooling is a methodologically appropriate design to study methylation variation in response to a given stimulus. Additionally, comparative analyses to publicly available databases confirm that MSAP is an effective tool for DNA methylation profiling when WGBS is not feasible, either due to lack of genomics/epigenomic data, or because of a large optimal sample size.

## Methods

### Plant material

A69Y, W23, B73 and Mo17 inbred lines were grown in the field where out-crosses were performed to produce **W23**/A69Y and **Mo17**/B73 F1 hybrids (the egg donor of the cross is underlined). B73 and Mo17 seeds were obtained from the Maize Genetics Cooperation Stock Center, while A69Y and W23 seeds were a kind gift from Dr. Angelo Viotti. For each genotype, seeds were harvested either at 15 days after pollination (DAP) or at maturity. Individual endosperms were dissected from immature seeds, whereas mature seeds were germinated for two weeks in the greenhouse to obtain leaf tissue. In both cases, tissue was derived from a single cob.

### MSAP and AFLP analysis

MSAP restriction digests, ligations and pre- and selective PCR reactions were performed as previously described [42]. For each sample, three independent MSAP reactions were performed; once reproducible, one sample was used for further analysis. *Eco*RI and *Hpa*II preselective primers were: 5′-AGACTGCGTACCAATTC-3′ and 5′-TCATGAGTCCTGCTCGG-3′, respectively. Selective primers were identical to preselective primers including additional 3′ nucleotides. *Eco*RI selective primers were: *Eco*RI-01 AGT, *Eco*RI-02 ACA, *Eco*RI-03 AGA, *Eco*RI-04 ACC; *Hpa*II selective primers were: *Hpa*II-02 TAGC, *Hpa*II03 CGAA, *Hpa*II-03A CGTT, *Hpa*II-04 AATT. An MSAP band was scored at variable if it showed variation between individual endosperms. Only well resolved MSAP bands were scored.

AFLP was conducted as previously described [42]. Pre-selective primers were complementary to core sequences of *Eco*RI and *Mse*1 adaptors including one selective nucleotide for both *Eco*RI (5′- GACTGCGTACCAATTCA) and *Mse*I (5′-GATGAGTCCTGAGTAAC) primers. *Eco*RI selective primers were: E31 AAA and E32 AAC; selective *Mse*I primers were M47 CAA, M48 CAC, M49 CAG, M50 CAT, M51 CCA.

### Isolation and analysis of MSAP fragments

MSAP bands were isolated from acrylamide gels as previously reported [42]. In maize, a total of 58 and 48 fragments that showed or lacked variation in methylation, respectively were isolated from B73 and Mo17 inbred lines. Following re-amplification, PCR products were cloned and sequenced in triplicate on both strands. Only fragments that were of the expected size, contained the appropriate selective primer sequences and represented a single DNA sequence were selected for further analysis. Blast analysis of variable and non-variable fragments was performed against the updated maize B73 RefGen_V4 (http://ensembl.gramene.org/Zea_mays/); methylation values of variable and non-variable *Hpa*II sites were recovered from publicly available methylation data [32].

### DNA extraction and Southern blot analysis

Extraction of genomic DNA, restriction enzyme digests and Southern blotting was performed as described previously [42].

### Quantification of CG- and CHG methylation in maize endosperm

The in vitro methyl-accepting assay using S-adenosyl-L-[methyl-^3^H] methionine ([^3^H]SAM) was performed exactly as previously described [50]. The rationale of the assay is that when using [^3^H]SAM as substrate, the amount of incorporated radioactivity is directly proportional to the extent of initial DNA hypomethylation. Reactions were carried out with 0.3–0.5 μg DNA for 3 h. In these conditions, incorporation of radioactivity is linearly proportional to DNA concentration and the reaction is carried out to completeness [50]. Raw data were converted into copies of unmethylated target per haploid genome considering a haploid maize and mouse genome content of ~2.5 and 3.5 pgs, respectively [75] using the formula: 2.5(D*N*_A_)/AS where D is total incorporated radioactivity (dpm), *N*_A_ is Avogadro’s number, A is specific activity (dpm/mole), S is the amount of substrate DNA (pg).

### RNA extraction and RT-PCR analysis

RNA was extracted with TRIzol®Reagent (Cat. 15596-026 Life Technologies) according to the manufacturer’s instructions and treated with DNAseI (Turbo DNA-free kit, Ambion cat. AM1907). RNA quality was assessed by 1% agarose gel electrophoresis and quantified using a Nanodrop-1000 spectrophotometer. For RT-PCR analysis, cDNA was synthesized from 1 μg of total RNA (SuperScript III kit, Invitrogen). Subsequently, 1/20 reaction volume was used in a standard PCR reaction with gene-specific primers. Primer specificity was confirmed by sequence analysis.

### Statistical analysis

Multiple comparisons of MSAP data across hybrid and inbred lines were performed with the Kruskal-Wallis test. A student T-test for two independent means was used for comparisons of expression profiles between leaf and endosperm tissues.

## Additional files

Additional file 1: Table S1. Extent of ii-MV in hybrid crosses and inbred maize lines. (DOCX 60 kb)
Additional file 2: Figure S1. Southern blot analysis of variable and non-variable fragments in the A69Y inbred line. **a**) DNA pooled from 14 day-old leaves (L) or endosperms (E) harvested 15 DAP were digested and probed with variable (v) and non-variable (n) MSAP fragments as indicated; arrows indicate hybridization to endosperm-specific bands; **b**) individual endosperms were digested and probed as indicated; arrows indicate bands that show ii-MV in a CG and CHG context. (PPTX 381 kb)
Additional file 3: Sequence of variable (v) and non-variable (n) MSAP fragments that mapped to unique regions of the B73 genome. Sequence data of isolated MSAP fragments that showed or lacked ii-MV (v and n, respectively). (TXT 8 kb)
Additional file 4: Figure S2. Comparison of MSAP and WGBS data. **a**) schematic representation of non-variable and variable MSAP profiles and the deduced methylation states of each *Hpa*II sites. Black bars and stippled white bars indicate presence and absence of an MSAP fragment, respectively; filled and empty circles indicate a methylated and unmethylated *Hpa*II site, respectively. The percentage of MSAP fragments representing each profile and the relative methylation state of *Hpa*II sites in leaf compared to endosperm is indicated right and left, respectively, of MSAP profiles; L ~ E indicates a similar methylation state of the *Hpa*II site in leaf and endosperm; L > E and L < E indicate *Hpa*II sites that are more or less methylated, respectively, in leaf relative to endosperm; **b** and **c**) comparison of predicted MSAP methylation states of non-variable and variable *Hpa*II sites to the CG or CHG methylation values obtained from WGBS of B73 (B) and Mo17 (M) leaf tissue [32]; + and – indicates a predicted methylated or unmethylated state, respectively; Y-axis indicates methylation levels between 0 and 1 (0 and 100% methylation, respectively). (PPTX 97 kb)
Additional file 5: Figure S3. CG and CHG methylation in leaf tissue of the 20 kb genomic regions surrounding non-variable or variable *Hpa*II sites. The Y-axis indicates leaf DNA methylation levels between 0 and 1 (0 and 100% methylation, respectively) obtained from WGBS of B73 and Mo17 leaf tissue [32]; arrowheads indicate positions of variable and non-variable *Hpa*II sites. (PPTX 1259 kb)
Additional file 6: Table S2. Blast analysis of variable and non-variable fragments. (PDF 55 kb)
Additional file 7: Figure S4. Classification of repetitive elements that were closest to a variable and non-variable *Hpa*II site. **a**) percentage of tandem repeats, class I and class II TEs; **b**) percentage of class I and class II TE superfamilies. (PPTX 52 kb)

## Abbreviations

([^3^H]SAM): S-adenosyl-L-[methyl-^3^H] methionine
AFLP: Amplified Fragment Length Polymorphism
DAP: Days after pollination
ii-MV: Inter-individual methylation variation
MSAP: Methylation Sensitive Amplified Polymorphism
n: Non-variable
tDMRs: Tissue-specific differentially methylated regions
TEs: Transposable elements
v: Variable

## References

[1] Gruenbaum Y, Naveh-Many T, Cedar H, Razin A (1981). Sequence specificity of methylation in higher plant DNA. Nature.

[2] Gruenbaum Y, Cedar H, Razin A (1982). Substrate and sequence specificity of a eukaryotic DNA methylase. Nature.

[3] Meyer P, Niedenhof I, ten Lohuis M (1994). Evidence for cytosine methylation of non-symmetrical sequences in transgenic Petunia hybrida. EMBO J.

[4] Cao X, Springer NM, Muszynski MG, Phillips RL, Kaeppler S, Jacobsen SE (2000). Conserved plant genes with similarity to mammalian de novo DNA methyltransferases. Proc Natl Acad Sci U S A.

[5] Bartee L, Malagnac F, Bender J (2001). Arabidopsis cmt3 chromomethylase mutations block non-CG methylation and silencing of an endogenous gene. Genes Dev.

[6] Lindroth AM, Cao X, Jackson JP, Zilberman D, McCallum CM, Henikoff S (2001). Requirement of CHROMOMETHYLASE3 for maintenance of CpXpG methylation. Science.

[7] Kishimoto N, Sakai H, Jackson J, Jacobsen SE, Meyerowitz EM, Dennis ES (2001). Site specificity of the Arabidopsis METI DNA methyltransferase demonstrated through hypermethylation of the superman locus. Plant Mol Biol.

[8] Papa CM, Springer NM, Muszynski MG, Meeley R, Kaeppler SM (2001). Maize chromomethylase Zea methyltransferase2 is required for CpNpG methylation. Plant Cell.

[9] Stroud H, Greenberg MVC, Feng S, Bernatavichute YV, Jacobsen SE (2013). Comprehensive analysis of silencing mutants reveals complex regulation of the Arabidopsis methylome. Cell.

[10] Li Q, Eichten SR, Hermanson PJ, Zaunbrecher VM, Song J, Wendt J (2014). Genetic perturbation of the maize methylome. Plant Cell.

[11] Cao X, Jacobsen SE (2002). Role of the arabidopsis DRM methyltransferases in de novo DNA methylation and gene silencing. Curr Biol.

[12] Wassenegger M, Heimes S, Riedel L, Sänger HL (1994). RNA-directed de novo methylation of genomic sequences in plants. Cell.

[13] Matzke MA, Mosher RA (2014). RNA-directed DNA methylation: an epigenetic pathway of increasing complexity. Nat Rev Genet.

[14] Zhang X, Yazaki J, Sundaresan A, Cokus S, Chan SW-L, Chen H (2006). Genome-wide high-resolution mapping and functional analysis of DNA methylation in arabidopsis. Cell.

[15] Zilberman D, Gehring M, Tran RK, Ballinger T, Henikoff S (2007). Genome-wide analysis of Arabidopsis thaliana DNA methylation uncovers an interdependence between methylation and transcription. Nat Genet.

[16] Wang X, Elling AA, Li X, Li N, Peng Z, He G (2009). Genome-wide and organ-specific landscapes of epigenetic modifications and their relationships to mRNA and small RNA transcriptomes in maize. Plant Cell.

[17] He G, Zhu X, Elling AA, Chen L, Wang X, Guo L (2010). Global epigenetic and transcriptional trends among two rice subspecies and their reciprocal hybrids. Plant Cell.

[18] Zemach A, McDaniel IE, Silva P, Zilberman D (2010). Genome-wide evolutionary analysis of eukaryotic DNA methylation. Science.

[19] Li X, Zhu J, Hu F, Ge S, Ye M, Xiang H (2012). Single-base resolution maps of cultivated and wild rice methylomes and regulatory roles of DNA methylation in plant gene expression. BMC Genomics.

[20] Song Q-X, Lu X, Li Q-T, Chen H, Hu X-Y, Ma B (2013). Genome-wide analysis of DNA methylation in soybean. Mol Plant.

[21] Gardiner L-J, Quinton-Tulloch M, Olohan L, Price J, Hall N, Hall A (2015). A genome-wide survey of DNA methylation in hexaploid wheat. Genome Biol.

[22] Kim KD, El Baidouri M, Abernathy B, Iwata-Otsubo A, Chavarro C, Gonzales M (2015). A Comparative Epigenomic Analysis of Polyploidy-Derived Genes in Soybean and Common Bean. Plant Physiol.

[23] Wang P, Xia H, Zhang Y, Zhao S, Zhao C, Hou L (2015). Genome-wide high-resolution mapping of DNA methylation identifies epigenetic variation across embryo and endosperm in Maize (Zea may). BMC Genomics.

[24] Chandler VL, Walbot V (1986). DNA modification of a maize transposable element correlates with loss of activity. Proc Natl Acad Sci U S A.

[25] Bestor TH (1990). DNA methylation: evolution of a bacterial immune function into a regulator of gene expression and genome structure in higher eukaryotes. Philos Trans R Soc Lond B Biol Sci.

[26] Vaughn MW, Tanurdzić M, Lippman Z, Jiang H, Carrasquillo R, Rabinowicz PD (2007). Epigenetic natural variation in Arabidopsis thaliana. PLoS Biol.

[27] Zhang X, Shiu S-H, Shiu S, Cal A, Borevitz JO (2008). Global analysis of genetic, epigenetic and transcriptional polymorphisms in Arabidopsis thaliana using whole genome tiling arrays. PLoS Genet.

[28] Becker C, Hagmann J, Müller J, Koenig D, Stegle O, Borgwardt K (2011). Spontaneous epigenetic variation in the Arabidopsis thaliana methylome. Nature.

[29] Eichten SR, Swanson-Wagner RA, Schnable JC, Waters AJ, Hermanson PJ, Liu S (2011). Heritable epigenetic variation among maize inbreds. PLoS Genet.

[30] Schmitz RJ, Schultz MD, Lewsey MG, O’Malley RC, Urich MA, Libiger O (2011). Transgenerational epigenetic instability is a source of novel methylation variants. Science.

[31] Eichten SR, Briskine R, Song J, Li Q, Swanson-Wagner R, Hermanson PJ (2013). Epigenetic and genetic influences on DNA methylation variation in maize populations. Plant Cell.

[32] Regulski M, Lu Z, Kendall J, Donoghue MTA, Reinders J, Llaca V (2013). The maize methylome influences mRNA splice sites and reveals widespread paramutation-like switches guided by small RNA. Genome Res.

[33] Schmitz RJ, He Y, Valdés-López O, Khan SM, Joshi T, Urich MA (2013). Epigenome-wide inheritance of cytosine methylation variants in a recombinant inbred population. Genome Res.

[34] Zhang M, Xie S, Dong X, Zhao X, Zeng B, Chen J (2014). Genome-wide high resolution parental-specific DNA and histone methylation maps uncover patterns of imprinting regulation in maize. Genome Res.

[35] Widman N, Feng S, Jacobsen SE, Pellegrini M (2014). Epigenetic differences between shoots and roots in Arabidopsis reveals tissue-specific regulation. Epigenetics.

[36] Hagmann J, Becker C, Müller J, Stegle O, Meyer RC, Wang G (2015). Century-scale methylome stability in a recently diverged Arabidopsis thaliana lineage. PLoS Genet.

[37] Cervera MT, Ruiz-García L, Martínez-Zapater JM (2002). Analysis of DNA methylation in Arabidopsis thaliana based on methylation-sensitive AFLP markers. Mol Genet Genomics.

[38] Zhang MS, Yan HY, Zhao N, Lin XY, Pang JS, Xu KZ (2007). Endosperm-specific hypomethylation, and meiotic inheritance and variation of DNA methylation level and pattern in sorghum (Sorghum bicolor L.) inter-strain hybrids. Theor Appl Genet.

[39] Zhao X, Chai Y, Liu B (2007). Epigenetic inheritance and variation of DNA methylation level and pattern in maize intra-specific hybrids. Plant Sci.

[40] Wang H, Chai Y, Chu X, Zhao Y, Wu Y, Zhao J (2009). Molecular characterization of a rice mutator-phenotype derived from an incompatible cross-pollination reveals transgenerational mobilization of multiple transposable elements and extensive epigenetic instability. BMC Plant Biol.

[41] Verhoeven KJF, Jansen JJ, van Dijk PJ, Biere A (2010). Stress-induced DNA methylation changes and their heritability in asexual dandelions. New Phytol.

[42] Lauria M, Rupe M, Guo M, Kranz E, Pirona R, Viotti A (2004). Extensive maternal DNA hypomethylation in the endosperm of Zea mays. Plant Cell.

[43] Gehring M, Bubb KL, Henikoff S (2009). Extensive demethylation of repetitive elements during seed development underlies gene imprinting. Science.

[44] Hsieh T-F, Ibarra CA, Silva P, Zemach A, Eshed-Williams L, Fischer RL (2009). Genome-wide demethylation of Arabidopsis endosperm. Science.

[45] Zemach A, Kim MY, Silva P, Rodrigues JA, Dotson B, Brooks MD (2010). Local DNA hypomethylation activates genes in rice endosperm. Proc Natl Acad Sci U S A.

[46] Xu W, Dai M, Li F, Liu A (2014). Genomic imprinting, methylation and parent-of-origin effects in reciprocal hybrid endosperm of castor bean. Nucleic Acids Res.

[47] Xing M-Q, Zhang Y-J, Zhou S-R, Hu W-Y, Wu X-T, Ye Y-J (2015). Global analysis reveals the crucial roles of DNA methylation during rice seed development. Plant Physiol.

[48] Vos P, Hogers R, Bleeker M, Reijans M, van de Lee T, Hornes M (1995). AFLP: a new technique for DNA fingerprinting. Nucleic Acids Res.

[49] Reyna-López GE, Simpson J, Ruiz-Herrera J (1997). Differences in DNA methylation patterns are detectable during the dimorphic transition of fungi by amplification of restriction polymorphisms. Mol Gen Genet.

[50] Schmitt F, Oakeley EJ, Jost JP (1997). Antibiotics induce genome-wide hypermethylation in cultured Nicotiana tabacum plants. J Biol Chem.

[51] Warnecke PM, Stirzaker C, Melki JR, Millar DS, Paul CL, Clark SJ (1997). Detection and measurement of PCR bias in quantitative methylation analysis of bisulphite-treated DNA. Nucleic Acids Res.

[52] Sekhon RS, Lin H, Childs KL, Hansey CN, Buell CR, de Leon N (2011). Genome-wide atlas of transcription during maize development. Plant J.

[53] Oey H, Isbel L, Hickey P, Ebaid B, Whitelaw E (2015). Genetic and epigenetic variation among inbred mouse littermates: identification of inter-individual differentially methylated regions. Epigenetics and chromatin.

[54] Qu W, Tsukahara T, Nakamura R, Yurino H, Hashimoto S, Tsuji S (2016). Assessing cell-to-cell DNA methylation variability on individual long reads. Sci Rep.

[55] Saze H, Shiraishi A, Miura A, Kakutani T (2008). Control of genic DNA methylation by a jmjC domain-containing protein in Arabidopsis thaliana. Science.

[56] Miura A, Nakamura M, Inagaki S, Kobayashi A, Saze H, Kakutani T (2009). An Arabidopsis jmjC domain protein protects transcribed genes from DNA methylation at CHG sites. EMBO J.

[57] Yaari R, Noy-Malka C, Wiedemann G, Auerbach Gershovitz N, Reski R, Katz A (2015). DNA METHYLTRANSFERASE 1 is involved in mCG and mCCG DNA methylation and is essential for sporophyte development in Physcomitrella patens. Plant Mol Biol.

[58] Bewick AJ, Ji L, Niederhuth CE, Willing E-M, Hofmeister BT, Shi X (2016). On the origin and evolutionary consequences of gene body DNA methylation. Proc Natl Acad Sci.

[59] Lister R, O’Malley RC, Tonti-Filippini J, Gregory BD, Berry CC, Millar AH (2008). Highly integrated single-base resolution maps of the epigenome in Arabidopsis. Cell.

[60] Lauria M, Piccinini S, Pirona R, Lund G, Viotti A, Motto M (2014). Epigenetic variation, inheritance, and parent-of-origin effects of cytosine methylation in maize (Zea mays). Genetics Genetics.

[61] Eichten SR, Springer NM (2015). Minimal evidence for consistent changes in maize DNA methylation patterns following environmental stress. Front Plant Sci.

[62] Secco D, Wang C, Shou H, Schultz MD, Chiarenza S, Nussaume L, et al. Stress induced gene expression drives transient DNA methylation changes at adjacent repetitive elements. Elife. 2015;4:e09343. Available from: http://www.ncbi.nlm.nih.gov/pubmed/26196146 [cited 2016 Jun 19]. 10.7554/eLife.09343 PMC4534844 26196146

[63] Wibowo A, Becker C, Marconi G, Durr J, Price J, Hagmann J, et al. Hyperosmotic stress memory in Arabidopsis is mediated by distinct epigenetically labile sites in the genome and is restricted in the male germline by DNA glycosylase activity. Elife. 2016;5:e13546. Available from: http://elifesciences.org/lookup/doi/10.7554/eLife.13546 [cited 2016 Jul 4]. 10.7554/eLife.13546 PMC4887212 27242129

[64] Morgan HD, Sutherland HG, Martin DI, Whitelaw E (1999). Epigenetic inheritance at the agouti locus in the mouse. Nat Genet.

[65] Reiss D, Zhang Y, Rouhi A, Reuter M, Mager DL (2010). Variable DNA methylation of transposable elements: the case study of mouse Early Transposons. Epigenetics.

[66] Rangwala SH, Elumalai R, Vanier C, Ozkan H, Galbraith DW, Richards EJ (2006). Meiotically stable natural epialleles of sadhu, a novel arabidopsis retroposon. PLoS Genet.

[67] Sandovici I, Kassovska-Bratinova S, Loredo-Osti JC, Leppert M, Suarez A, Stewart R (2005). Interindividual variability and parent of origin DNA methylation differences at specific human Alu elements. Hum Mol Genet.

[68] Luff B, Pawlowski L, Bender J (1999). An inverted repeat triggers cytosine methylation of identical sequences in Arabidopsis. Mol Cell.

[69] Melquist S, Luff B, Bender J (1999). Arabidopsis PAI gene arrangements, cytosine methylation and expression. Genetics.

[70] Martin A, Troadec C, Boualem A, Rajab M, Fernandez R, Morin H (2009). A transposon-induced epigenetic change leads to sex determination in melon. Nature.

[71] Hollister JD, Smith LM, Guo Y-L, Ott F, Weigel D, Gaut BS (2011). Transposable elements and small RNAs contribute to gene expression divergence between Arabidopsis thaliana and Arabidopsis lyrata. Proc Natl Acad Sci U S A.

[72] Durand S, Bouché N, Perez Strand E, Loudet O, Camilleri C (2012). Rapid establishment of genetic incompatibility through natural epigenetic variation. Curr Biol.

[73] Eichten SR, Ellis NA, Makarevitch I, Yeh C-T, Gent JI, Guo L (2012). Spreading of heterochromatin is limited to specific families of maize retrotransposons. PLoS Genet.

[74] Zhang J, Liu Y, Xia E-H, Yao Q-Y, Liu X-D, Gao L-Z (2015). Autotetraploid rice methylome analysis reveals methylation variation of transposable elements and their effects on gene expression. Proc Natl Acad Sci U S A.

[75] Arumuganathan K, Earle ED (1991). Nuclear DNA content of some important plant species. Plant Mol Biol Report.

