# Supplementary material for: Inter-individual variation in DNA methylation is largely restricted to tissue-specific differentially methylated regions in maize
Source: BMC Plant Biol. 2017 Feb 23;17:52. doi: 10.1186/s12870-017-0997-3 (PMC5324254; doi:10.1186/s12870-017-0997-3)
Supplement: Additional file 1: Table S1. — Extent of ii-MV in hybrid crosses and inbred maize lines. (DOCX 60 kb) [file 12870_2017_997_MOESM1_ESM.docx]

| **Table S1. Inter-individual variation in methylation in hybrid and inbred maize lines.**  and inbrec miaze and inbred endosperms progeny | | | | |
| --- | --- | --- | --- | --- |
| Cross/inbred | Tissue | Total^*^ | Variable^†^ | % variable |
| W23/A69Y^‡^ | Endosperm | 526 | 69 | 13 |
|  | Leaf | 440 | 14 | 3 |
| Mo17/B73^‡^ | Endosperm | 662 | 85 | 13 |
|  | Leaf | 528 | 20 | 4 |
| A69Y^§^ | Endosperm | 301 | 33 | 11 |
| ^*^sum of MSAP fragments that lacked or showed variation in DNA methylation.  ^†^MSAP fragments that show variation in individual MSAP profiles.  ^‡^variation was scored of 10 individual endosperms using 12 selective primer combinations.  ^§^variation was scored of 8 individual endosperms using 5 selective primer combinations. | | | | |
