# Supplementary material for: Inter-individual variation in DNA methylation is largely restricted to tissue-specific differentially methylated regions in maize
Source: BMC Plant Biol. 2017 Feb 23;17:52. doi: 10.1186/s12870-017-0997-3 (PMC5324254; doi:10.1186/s12870-017-0997-3)
Supplement: Additional file 2: Figure S1. — Southern blot analysis of variable and non-variable fragments in the A69Y inbred line. a) DNA pooled from 14 day-old leaves (L) or endosperms (E) harvested 15 DAP were digested and probed with variable (v) and non-variable (n) MSAP fragments as indicated; arrows indicate hybridization to endosperm-specific bands; b) individual endosperms were digested and probed as indicated; arrows indicate bands that show ii-MV in a CG and CHG context. (PPTX 381 kb) [file 12870_2017_997_MOESM2_ESM.pptx]

## Slide 1
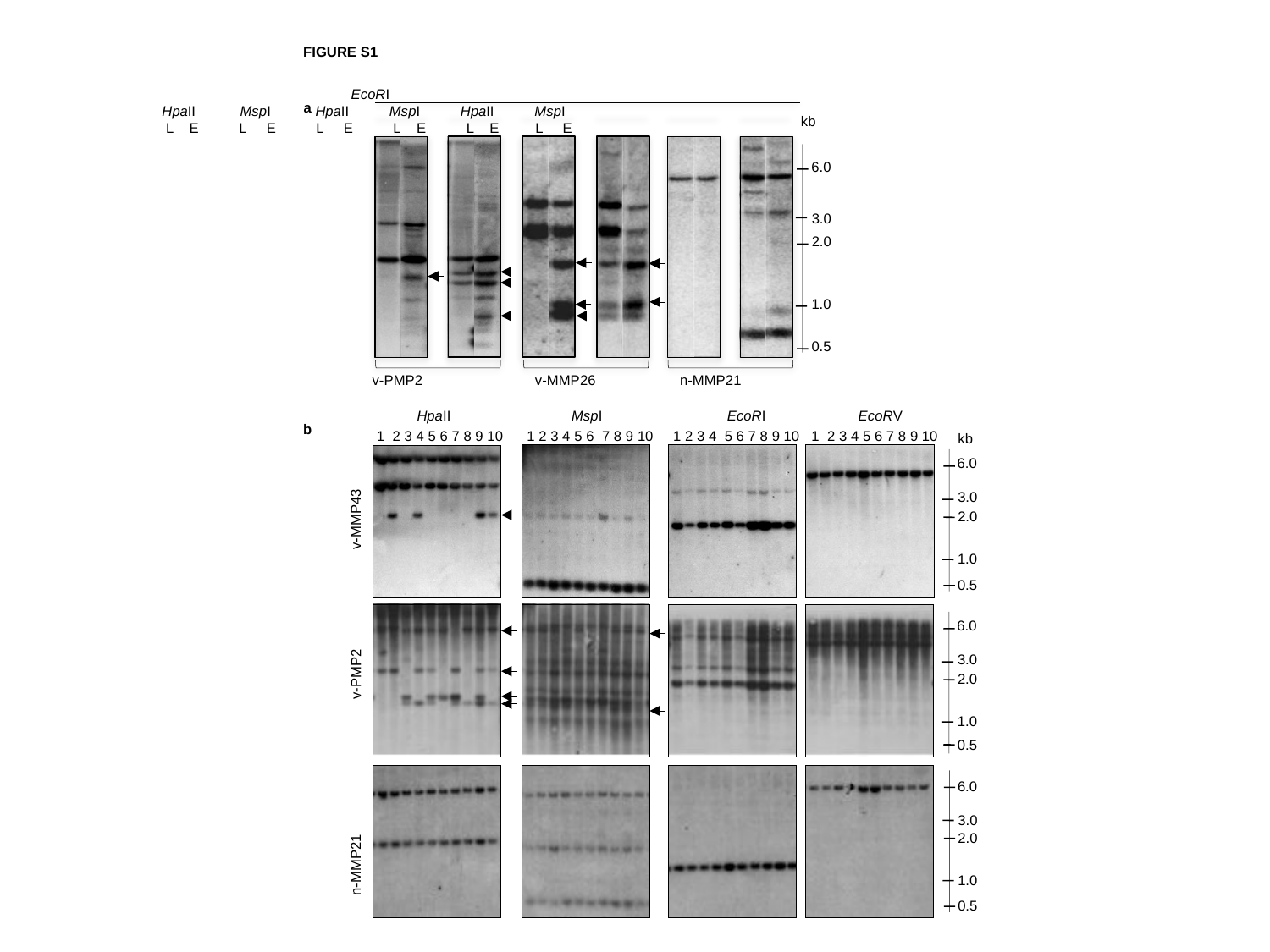

FIGURE S1
 EcoRI
HpaII MspI HpaII MspI HpaII MspI
 L E L E L E L E L E L E
kb
6.0
3.0
2.0
1.0
0.5
v-PMP2 v-MMP26 n-MMP21
a
 HpaII MspI EcoRI EcoRV
1 2 3 4 5 6 7 8 9 10 1 2 3 4 5 6 7 8 9 10 1 2 3 4 5 6 7 8 9 10 1 2 3 4 5 6 7 8 9 10
b
kb
6.0
3.0
2.0
v-MMP43
1.0
0.5
6.0
3.0
v-PMP2
2.0
1.0
0.5
6.0
3.0
n-MMP21
2.0
1.0
0.5
