# Supplementary material for: Inter-individual variation in DNA methylation is largely restricted to tissue-specific differentially methylated regions in maize
Source: BMC Plant Biol. 2017 Feb 23;17:52. doi: 10.1186/s12870-017-0997-3 (PMC5324254; doi:10.1186/s12870-017-0997-3)
Supplement: Additional file 4: Figure S2. — Comparison of MSAP and WGBS data. a) schematic representation of non-variable and variable MSAP profiles and the deduced methylation states of each HpaII sites. Black bars and stippled white bars indicate presence and absence of an MSAP fragment, respectively; filled and empty circles indicate a methylated and unmethylated HpaII site, respectively. The percentage of MSAP fragments representing each profile and the relative methylation state of HpaII sites in leaf compared to endosperm is indicated right and left, respectively, of MSAP profiles; L ~ E indicates a similar methylation state of the HpaII site in leaf and endosperm; L > E and L < E indicate HpaII sites that are more or less methylated, respectively, in leaf relative to endosperm; b and c) comparison of predicted MSAP methylation states of non-variable and variable HpaII sites to the CG or CHG methylation values obtained from WGBS of B73 (B) and Mo17 (M) leaf tissue [32]; + and – indicates a predicted methylated or unmethylated state, respectively; Y-axis indicates methylation levels between 0 and 1 (0 and 100% methylation, respectively). (PPTX 97 kb) [file 12870_2017_997_MOESM4_ESM.pptx]

## Slide 1
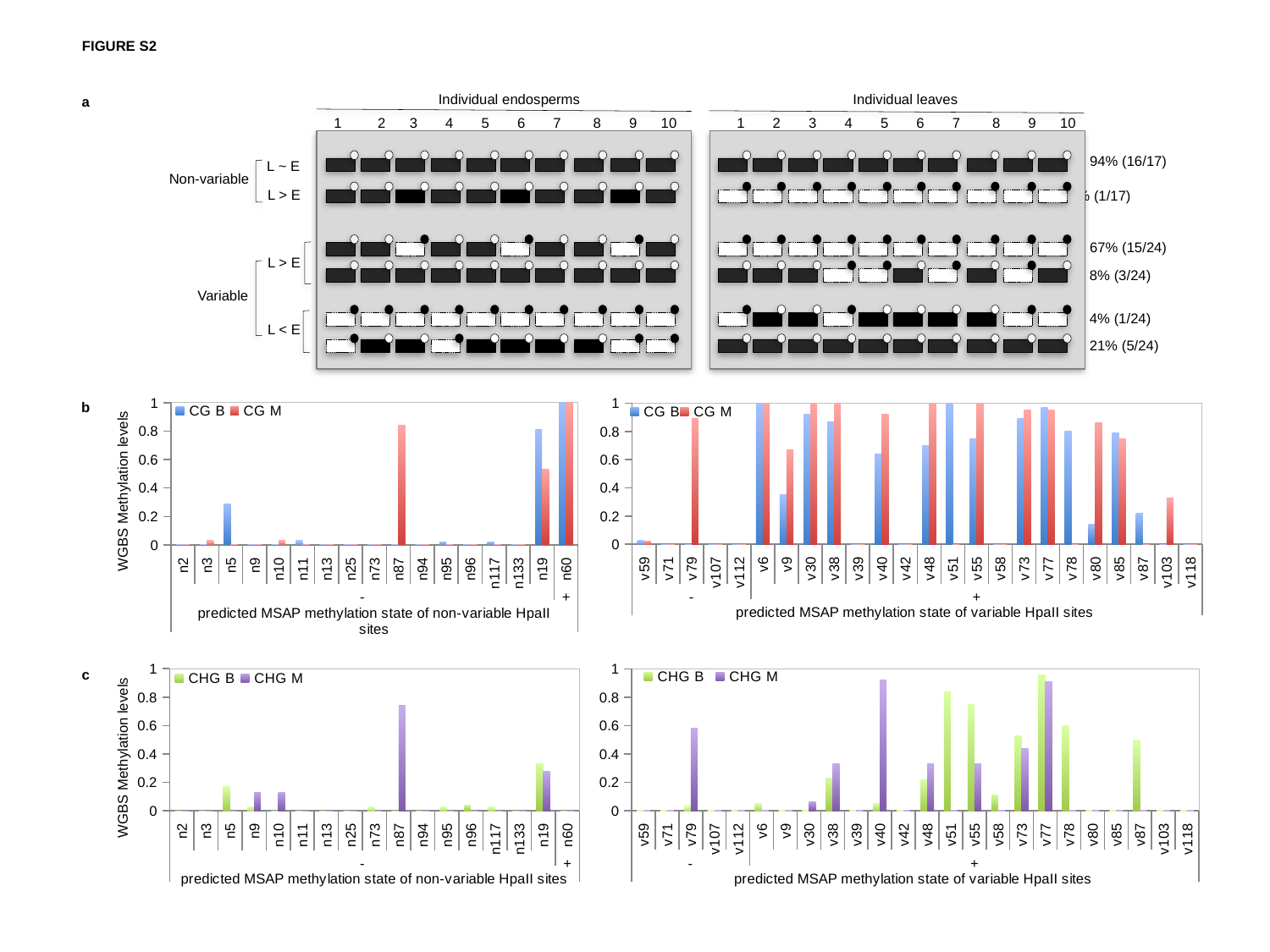

FIGURE S2
 Individual endosperms Individual leaves
1 2 3 4 5 6 7 8 9 10 1 2 3 4 5 6 7 8 9 10
a
94% (16/17)
L ~ E
Non-variable
L > E
6% (1/17)
67% (15/24)
L > E
8% (3/24)
Variable
4% (1/24)
L < E
21% (5/24)
### Chart
| Category | CG B | CG M |
|---|---|---|
| v59 | 0.03 | 0.02 |
| v71 | 0.0 | 0.0 |
| v79 | 0.0 | 0.89 |
| v107 | 0.0 | 0.0 |
| v112 | 0.0 | 0.0 |
| v6 | 1.0 | 1.0 |
| v9 | 0.35 | 0.67 |
| v30 | 0.92 | 1.0 |
| v38 | 0.87 | 1.0 |
| v39 | 0.0 | 0.0 |
| v40 | 0.64 | 0.92 |
| v42 | 0.0 | 0.0 |
| v48 | 0.7 | 1.0 |
| v51 | 1.0 | 0.0 |
| v55 | 0.75 | 1.0 |
| v58 | 0.0 | 0.0 |
| v73 | 0.89 | 0.95 |
| v77 | 0.97 | 0.95 |
| v78 | 0.8 | 0.0 |
| v80 | 0.14 | 0.86 |
| v85 | 0.79 | 0.75 |
| v87 | 0.22 | 0.0 |
| v103 | 0.0 | 0.33 |
| v118 | 0.0 | 0.0 |
### Chart
| Category | CG B | CG M |
|---|---|---|
| n2 | 0.0 | 0.0 |
| n3 | 0.0 | 0.03 |
| n5 | 0.29 | 0.0 |
| n9 | 0.0 | 0.0 |
| n10 | 0.0 | 0.03 |
| n11 | 0.03 | 0.0 |
| n13 | 0.0 | 0.0 |
| n25 | 0.0 | 0.0 |
| n73 | 0.0 | 0.0 |
| n87 | 0.0 | 0.84 |
| n94 | 0.0 | 0.0 |
| n95 | 0.02 | 0.0 |
| n96 | 0.0 | 0.0 |
| n117 | 0.02 | 0.0 |
| n133 | 0.0 | 0.0 |
| n19 | 0.81 | 0.53 |
| n60 | 1.0 | 1.0 |b
WGBS Methylation levels
### Chart
| Category | CHG B | CHG M |
|---|---|---|
| v59 | 0.0 | 0.0 |
| v71 | 0.0 | 0.0 |
| v79 | 0.03 | 0.58 |
| v107 | 0.0 | 0.0 |
| v112 | 0.0 | 0.0 |
| v6 | 0.05 | 0.0 |
| v9 | 0.0 | 0.0 |
| v30 | 0.0 | 0.06 |
| v38 | 0.23 | 0.33 |
| v39 | 0.0 | 0.0 |
| v40 | 0.05 | 0.92 |
| v42 | 0.0 | 0.0 |
| v48 | 0.22 | 0.33 |
| v51 | 0.84 | 0.0 |
| v55 | 0.75 | 0.33 |
| v58 | 0.11 | 0.0 |
| v73 | 0.53 | 0.44 |
| v77 | 0.96 | 0.91 |
| v78 | 0.6 | 0.0 |
| v80 | 0.0 | 0.0 |
| v85 | 0.0 | 0.0 |
| v87 | 0.5 | 0.0 |
| v103 | 0.0 | 0.0 |
| v118 | 0.0 | 0.0 |
### Chart
| Category | CHG B | CHG M |
|---|---|---|
| n2 | 0.0 | 0.0 |
| n3 | 0.0 | 0.0 |
| n5 | 0.17 | 0.0 |
| n9 | 0.03 | 0.13 |
| n10 | 0.0 | 0.13 |
| n11 | 0.0 | 0.0 |
| n13 | 0.0 | 0.0 |
| n25 | 0.0 | 0.0 |
| n73 | 0.03 | 0.0 |
| n87 | 0.0 | 0.74 |
| n94 | 0.0 | 0.0 |
| n95 | 0.03 | 0.0 |
| n96 | 0.04 | 0.0 |
| n117 | 0.03 | 0.0 |
| n133 | 0.0 | 0.0 |
| n19 | 0.33 | 0.28 |
| n60 | 0.0 | 0.0 |c
WGBS Methylation levels
