# Supplementary material for: Inter-individual variation in DNA methylation is largely restricted to tissue-specific differentially methylated regions in maize
Source: BMC Plant Biol. 2017 Feb 23;17:52. doi: 10.1186/s12870-017-0997-3 (PMC5324254; doi:10.1186/s12870-017-0997-3)
Supplement: Additional file 5: Figure S3. — CG and CHG methylation in leaf tissue of the 20 kb genomic regions surrounding non-variable or variable HpaII sites. The Y-axis indicates leaf DNA methylation levels between 0 and 1 (0 and 100% methylation, respectively) obtained from WGBS of B73 and Mo17 leaf tissue [32]; arrowheads indicate positions of variable and non-variable HpaII sites. (PPTX 1259 kb) [file 12870_2017_997_MOESM5_ESM.pptx]

## Slide 1
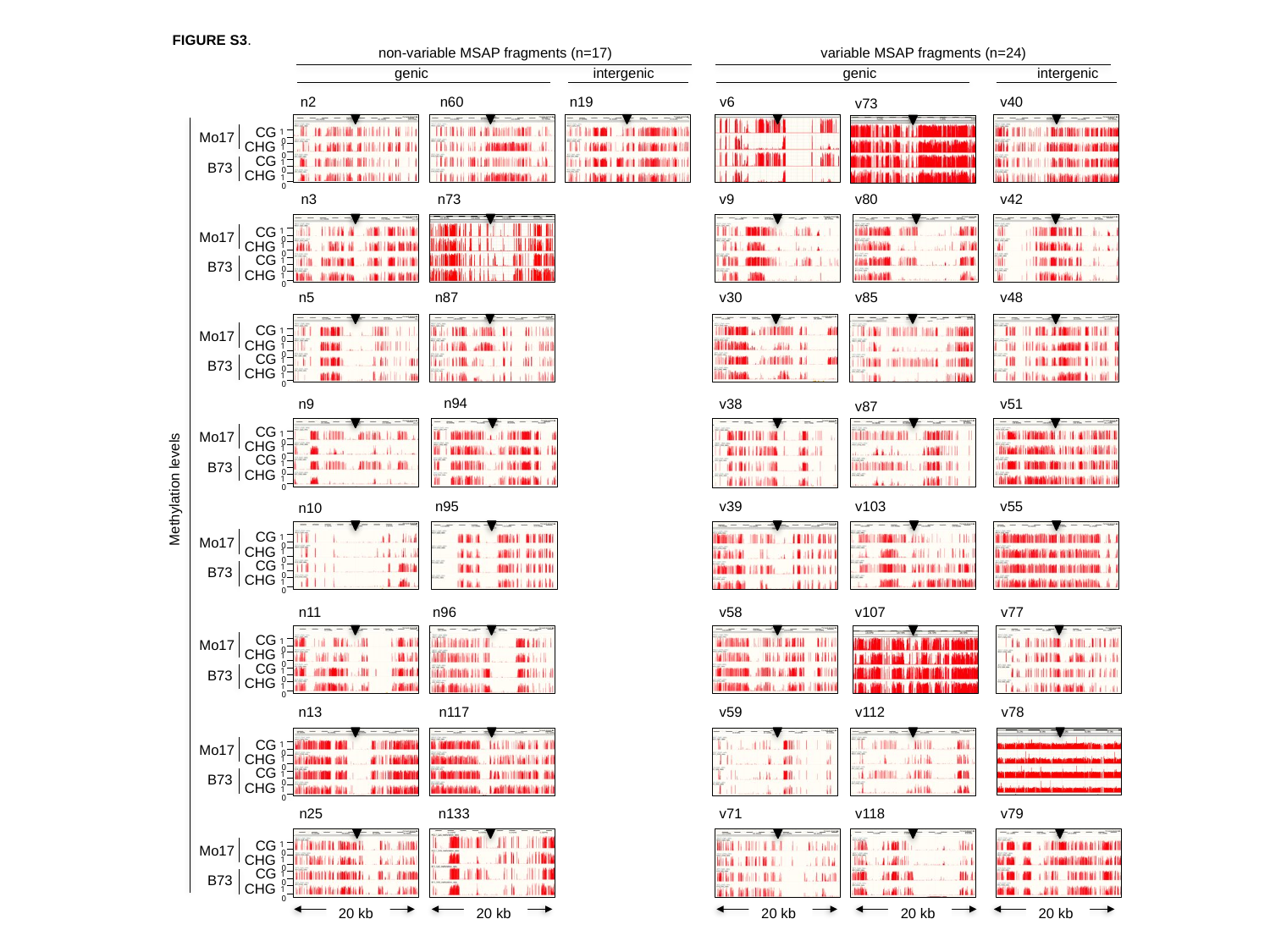

FIGURE S3.
non-variable MSAP fragments (n=17) variable MSAP fragments (n=24)
 genic intergenic genic intergenic
n2
n60
n19
v6
v40
v73
CG
CHG
Mo17
CG
CHG
B73
1
0
1
0
1
0
1
0
n3
n73
v9
v80
v42
n87
CG
CHG
Mo17
CG
CHG
B73
1
0
1
0
1
0
1
0
n5
n87
v30
v85
v48
CG
CHG
Mo17
CG
CHG
B73
1
0
1
0
1
0
1
0
n94
n9
v38
v51
v87
CG
CHG
Mo17
CG
CHG
B73
1
0
1
0
1
0
1
0
Methylation levels
n95
v39
v103
v55
n10
CG
CHG
Mo17
CG
CHG
B73
1
0
1
0
1
0
1
0
n11
n96
v58
v107
v77
CG
CHG
Mo17
CG
CHG
B73
1
0
1
0
1
0
1
0
n13
n117
v59
v112
v78
CG
CHG
Mo17
CG
CHG
B73
1
0
1
0
1
0
1
0
n25
n133
v71
v118
v79
CG
CHG
Mo17
CG
CHG
B73
1
0
1
0
1
0
1
0
20 kb
20 kb
20 kb
20 kb
20 kb
