# Supplementary material for: Inter-individual variation in DNA methylation is largely restricted to tissue-specific differentially methylated regions in maize
Source: BMC Plant Biol. 2017 Feb 23;17:52. doi: 10.1186/s12870-017-0997-3 (PMC5324254; doi:10.1186/s12870-017-0997-3)
Supplement: Additional file 6: Table S2. — Blast analysis of variable and non-variable fragments. (PDF 55 kb) [file 12870_2017_997_MOESM6_ESM.pdf]

Table S2: Blast analysis of variable (v) and non-variable (n) *Hpa*II sites located within genic regions.

	ID	Chr	Description	paralogue maize	Chr	Description	Orthologue (A.thaliana)
v6	GRMZM2G028190	8	Uncharacterized protein	GRMZM2G055578	3	Uncharacterized protein	T1G15280 CASC3/Barentsz eIF4AIII binding
v9	GRMZM2G097109	1	Uncharacterized protein	GRMZM2G412229	5	Uncharacterized protein	AT1G54520, unknown protein
v30	Zm00001d011952	8	Uncharacterized protein	Zm00001d042266	3		none
v38	Zm00001d017781	5	Zm00001d017781	Zm00001d010734	8		none
v39	GRMZM2G003172	7	ATP12 ATPase	none			AT5G40660 ATP12 protein-related
v58	GRMZM5G817886	2	Uncharacterized protein	GRMZM2G108125	10	Putative aminotransferase class III superfamily protein; Uncharacterized protein	none
v59	GRMZM5G817886	2	Uncharacterized protein	GRMZM2G108125	10	Putative aminotransferase class III superfamily protein;	none
v71	GRMZM2G068392	8	Uncharacterized protein	none			AT1G04830 Ypt/Rab-GAP domain of gyp1p superfamily protein
v73	GRMZM2G092465	6	Putative NAC domain transcription factor superfamily protein	none			NST1 Putative uncharacterized protein
v80	GRMZM2G062394	1	Serine/threonine-protein phosphatase	GRMZM2G108355	5	Serine/threonine-protein phosphatase	PP2A4 Serine/threonine-protein phosphatase PP2A-4 catalytic subunit
v85	GRMZM2G104254	5	Uncharacterized protein	GRMZM2G148323	1	Uncharacterized protein	B''EPSILON Probable serine/threonine protein phosphatase 2A regulatory subunit B''epsilon
v87	GRMZM2G042231	9	Uncharacterized protein	nono			TAF1 Transcription initiation factor TFIID subunit 1
v103	GRMZM2G124151	4	Uncharacterized protein	none		none	AT2G19160 Core-2/I-branching beta-1,6-N-acetylglucosaminyltransferase family protein
v107	GRMZM2G041060	4	Peptidyl-prolyl cis-trans isomerase	none			FKBP17-1 Peptidyl-prolyl cis-trans isomerase FKBP17-1, chloroplastic
v112	GRMZM2G179002	2	Uncharacterized protein	GRMZM5G868588	7	Palmitoyltransferase ZDHHC9	PAT07 Probable protein S-acyltransferase 7
v118	GRMZM2G107532	1	Uncharacterized protein	none			AT2G20240 Protein of unknown function (DUF3741)
n2	GRMZM2G028039	9	Uncharacterized protein	none			none
n3	GRMZM2G065259	9	Uncharacterized protein	none			none
n5	GRMZM2G038032	6	Guanine nucleotide-binding protein beta subunit-like protein	GRMZM2G040477	8	Guanine nucleotide-binding protein beta subunit-like protein	AT1G18080 RACK1A Receptor for activated C kinase 1A
n9	GRMZM2G150912	5	endoribonucleases	none			AT3G04480 endoribonucleases
n10	GRMZM2G052476	5	Uncharacterized protein	GRMZM5G843914	1	Uncharacterized protein	AT2G23700 Protein of unknown function, DUF547
n11	GRMZM2G029537	3	Uncharacterized protein	GRMZM2G168077	8	Uncharacterized protein	none
n13	GRMZM2G398854	6	Uncharacterized protein	none			AT5G60900 RLK1 receptor-like protein kinase 1
n25	GRMZM2G016250	6	60S ribosomal protein L27	none			AT2G32220 RPL27A 60S ribosomal protein L27-1
n60	Zm00001d043949	3	Uncharacterized protein	Zm00001d028875	1	Uncharacterized protein	Lon protease homolog
n73	GRMZM2G702599	4	Uncharacterized protein	none			none
n87	Zm00001d048179	9	Uncharacterized protein	none			none
n94	GRMZM2G107759	5	Uncharacterized protein	none			none
n95	GRMZM2G014356	4	Uncharacterized protein	GRMZM2G088543	5	Uncharacterized protein	none
n96	GRMZM2G074908	3	Uncharacterized protein	none			none
n117	GRMZM2G064626	4	Uncharacterized protein	GRMZM2G057717	5	Uncharacterized protein	AT2G44670 Protein of unknown function (DUF581)
n133	GRMZM2G180596	10	Uncharacterized protein	none			AT2G36020 HVA22J HVA22-like protein j
