# Supplementary material for: Inter-individual variation in DNA methylation is largely restricted to tissue-specific differentially methylated regions in maize
Source: BMC Plant Biol. 2017 Feb 23;17:52. doi: 10.1186/s12870-017-0997-3 (PMC5324254; doi:10.1186/s12870-017-0997-3)
Supplement: Additional file 7: Figure S4. — Classification of repetitive elements that were closest to a variable and non-variable HpaII site. a) percentage of tandem repeats, class I and class II TEs; b) percentage of class I and class II TE superfamilies. (PPTX 52 kb) [file 12870_2017_997_MOESM7_ESM.pptx]

## Slide 1
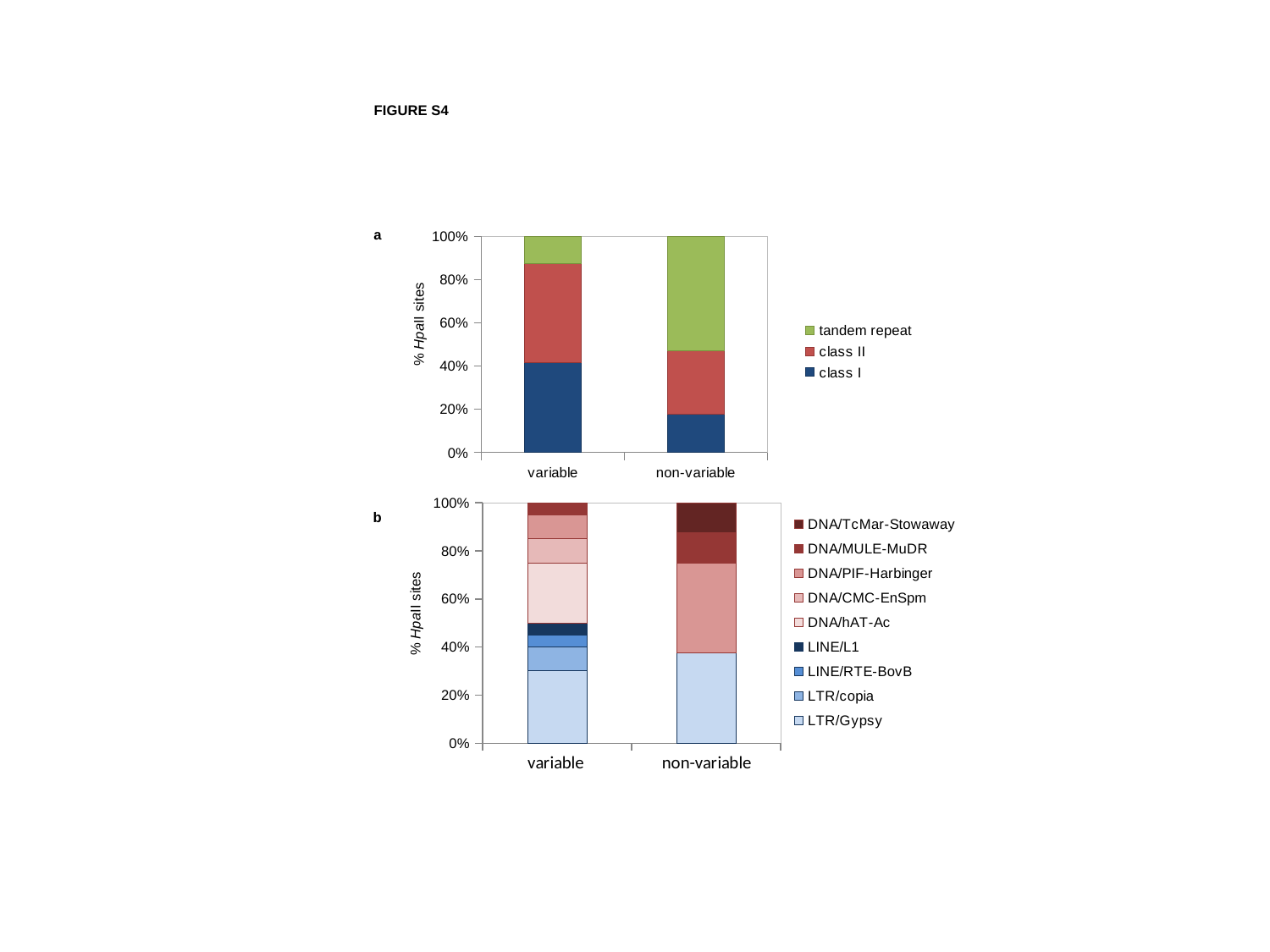

FIGURE S4
### Chart
| Category | class I | class II | tandem repeat |
|---|---|---|---|
| variable | 10.0 | 11.0 | 3.0 |
| non-variable | 3.0 | 5.0 | 9.0 |a
% HpaII sites
### Chart
| Category | LTR/Gypsy | LTR/copia | LINE/RTE-BovB | LINE/L1 | DNA/hAT-Ac | DNA/CMC-EnSpm | DNA/PIF-Harbinger | DNA/MULE-MuDR | DNA/TcMar-Stowaway |
|---|---|---|---|---|---|---|---|---|---|
| variable | 6.0 | 2.0 | 1.0 | 1.0 | 5.0 | 2.0 | 2.0 | 1.0 | 0.0 |
| non-variable | 3.0 | 0.0 | 0.0 | 0.0 | 0.0 | 0.0 | 3.0 | 1.0 | 1.0 |b
% HpaII sites
